# Ovarian mast cells migrate toward ovary-fimbria connection in neonatal MRL/MpJ mice

**DOI:** 10.1371/journal.pone.0196364

**Published:** 2018-04-23

**Authors:** Teppei Nakamura, Masataka Chihara, Osamu Ichii, Saori Otsuka-Kanazawa, Ken-ichi Nagasaki, Yaser Hosny Ali Elewa, Osamu Tatsumi, Yasuhiro Kon

**Affiliations:** 1 Laboratory of Anatomy, Department of Basic Veterinary Sciences, Division of Veterinary Medicine, Faculty of Veterinary Medicine, Hokkaido University, Sapporo, Hokkaido, Japan; 2 Section of Biological Science, Chitose Laboratory, Japan Food Research Laboratories, Chitose, Hokkaido, Japan; 3 Section of Biological Safety Research, Tama Laboratory, Japan Food Research Laboratories, Tama, Tokyo, Japan; 4 Department of Histology and Cytology, Faculty of Veterinary Medicine, Zagazig University, Zagazig, Egypt; Shanghai Ocean University, CHINA

## Abstract

MRL/MpJ mice have abundant ovarian mast cells (MCs) as compared with other strains at postnatal day 0 (P0); however, they sharply decrease after birth. These ovarian MCs, particularly beneath the ovarian surface epithelium (SE), which express mucosal MC (MMC) marker, might participate in early follicular development. This study investigated the changes in spatiotemporal distribution of MCs in the perinatal MRL/MpJ mouse ovaries. At P0 to P7, the MCs were densely localized to the ovary, especially their caudomedial region around the ovary-fimbria connection. The neonatal ovarian MCs showed intermediate characteristics of MMC and connective tissue MC (CTMC), and the latter phenotype became evident with aging. However, the expression ratio of the MMC to CTMC marker increased from P0 to P4 in the MRL/MpJ mouse ovary. Similarly, the ratio of MCs facing SE to total MC number increased with aging, although the number of ovarian MCs decreased, indicating the relative increase in MMC phenotypes in the early neonatal ovary. Neither proliferating nor apoptotic MCs were found in the MRL/MpJ mouse ovaries. The parenchymal cells surrounding MCs at ovary-fimbria connection showed similar molecular expression patterns (E-cadherin^+^/Foxl2^-^/Gata4^+^) as that of the ovarian surface epithelial cells. At P2, around the ovary-fimbria connection, c-kit^-^ immature oocytes formed clusters called nests, and some MCs localized adjacent to c-kit^-^ oocytes within the nests. These results indicated that in postnatal MRL/MpJ mice, ovarian MCs changed their distribution by migrating toward the parenchymal cells composing ovary-fimbria connection, which possessed similar characteristics to the ovarian surface epithelium. Thus, we elucidated the spatiotemporal alterations of the ovarian MCs in MRL/MpJ mice, and suggested their importance during the early follicular development by migrating toward the ovary-fimbria connection. MRL/MpJ mice would be useful to elucidate the relationship between neonatal immunity and reproductive systems.

## Introduction

Mast cells (MCs) are derived from the bone marrow and migrate into the local tissues where they mature depending on their microenvironments [1]. Mature MCs are classified into two subpopulations, namely, mucosal MCs (MMCs) and connective tissue MCs (CTMCs) [2]. In mice, MMCs are migratory cells, and possess chondroitin sulfate, stained with alcian blue (AB), and express mast cell protease 1 (Mcpt1) and Mcpt2. In contrast, CTMCs are characteristically non-migratory cells, and possess heparin and histamine, stained with safranin O (SO), and express Mcpt4, chymase 1 (Cma1), tryptase beta 2 (Tpsb2), tryptase alpha/beta 1 (Tpsab1), and carboxypeptidase A3 (Cpa3), but lack the MMC markers Mcpt1 and Mcpt2. MCs have plasticity to change their phenotypes depending on the microenvironments of tissues [3], and MCs possessing both MMC and CTMC phenotypes increase under certain circumstances [4–6]. After maturation, MCs not only act as sentinel cells in both innate and adaptive immunity [7], but also contribute to other physiology, such as cancer [8], obesity [9], and wound healing [3]. In addition, a recent study demonstrated that MCs contribute to female reproductive processes [10].

We have previously reported that the autoimmune disease models, MRL/MpJ and their mutant strain MRL/MpJ-*Fas*^*lpr/lpr*^ mice, have unique phenotypes in the reproductive organs, such as metaphase-specific apoptosis of meiotic spermatocytes [11–13], heat shock resistance of spermatocytes found in experimental cryptorchidism [14, 15], testicular calcification after transient scrotal heat stress [16, 17], existence of testicular oocytes in newborn males [18, 19], and development of ovarian cysts originating from the rete ovarii [20]. In addition, female MRL/MpJ-*Fas*^*lpr/lpr*^ mice show abnormal reproductive processes [21, 22], indicating that these mouse strains are useful models for reproductive immunology.

We have also demonstrated that the perinatal MRL/MpJ mouse ovaries possess unique phenotypes such as accelerated oocyte development [23] and presence of numerous MCs [24]. The appearance of ovarian MCs in neonatal MRL/MpJ mice is controlled by environmental factors as well as filial genetic factors, and the abundance and distribution of ovarian MCs are regulated by independent genetic elements coded by chromosome 8 [25]. The ovarian MCs possess CTMC phenotypes; however, the ones that localize beneath the ovarian surface epithelium (SE) express MMC marker, and the latter populations might contribute to the early follicular development in neonatal MRL/MpJ mice [24], indicating that the distribution of the ovarian MCs is crucial for reproductive functions. Therefore, we examined the spatial distribution changes of MCs in the female reproductive organs of perinatal MRL/MpJ mice.

## Materials and methods

### Animals and ethical statement

This study was approved by the Institutional Animal Care and Use Committee convened at the Graduate School of Veterinary Medicine, Hokkaido University (approval number: 13–0086). For all the experiments, the investigators adhered to the Guide for the Care and Use of Laboratory Animals of Hokkaido University, Graduate School of Veterinary Medicine (approved by the Association for the Assessment and Accreditation of Laboratory Animal Care International).

MRL/MpJ and C57BL/6N mice purchased from Japan SLC (Hamamatsu, Shizuoka, Japan) were used in this study. The mice were housed in plastic cages at 20 to 26°C under a 12 hour light/dark cycle, and had free access to a commercial diets and water. Timed mating was established by housing females with males overnight. At noon of the following day, females were checked for the presence of a vaginal plug, and the embryos were recorded as embryonic day 0.5 (E0.5).

### Whole mount staining of reproductive organs

After euthanasia with isoflurane, the female reproductive organs aged from postnatal day 0 (P0) to P4 were collected and fixed with 4% paraformaldehyde overnight. The ovaries were dehydrated with methanol, bleached with 3% H_2_O_2_ in methanol for 10 min, dehydrated with ethanol, defatted with xylene for 1 h, rehydrated with ethanol, acidified with 0.5 M hydrochloric acid, stained with alcian blue/safranin O (AB/SO) for 60 min [26], rinsed with 0.5 M hydrochloric acid, and penetrated with graded glycerol. MC distribution was examined by confocal microscopy (Keyence, Osaka, Japan) and stereo zoom microscopy (Zeiss, Oberkochen, Germany).

### Histology

The ovaries and skin of MRL/MpJ mice aged from E17.5 to P7 were fixed with 4% paraformaldehyde overnight, embedded in paraffin, and cut into 3-μm-thick sections. For the ovarian sections, we prepared transverse sections of the ovary by without detaching them from the body to make similar structural orientation. The sections were stained with 1% toluidine blue (TB) in 70% ethanol for 30 min, AB/SO for 30 min, or AB (pH 1.0)/PAS/hematoxylin. For the TB sections, the number of metachromatic MCs per total area was measured as the MC density (cells/mm^2^) [24, 25]. For quantification of the ovarian MCs, transverse sections of the ovaries were prepared at 20-μm intervals, and distributed equally among caudal, middle, and cranial regions. The ratio of the number of MCs facing the ovarian SE to those in the total ovarian area was calculated as SEMC ratio [25].

### Immunostaining

MRL/MpJ mice were subcutaneously injected with 100 mg/kg of 5-bromo-2-deoxyuridine (BrdU) 1 h before sampling. The ovaries of MRL/MpJ mice at P0 to P4 were collected and the ovarian sections were prepared as described above. Immunostaining was performed to detect BrdU, single stranded DNA (ssDNA), E-cadherin, Foxl2, Gata4, mast cell tryptase (CTMC marker), DEAD (Asp-Glu-Ala-Asp) box polypeptide 4 (DDX4, oocyte marker), and c-kit. The details of the procedures are listed in Table 1. Briefly, the deparaffinized sections were heated for antigen retrieval, and incubated with primary antibodies and secondary antibodies according to a previously published method [24]. For immunohistochemistry, the color was developed using 3,3ʹ-diaminobenzidine tetrahydrochloride-H_2_O_2_ solution. The sections were counterstained with AB (pH 1.0) and hematoxylin. The fluorescent signals were detected using a confocal microscope (Zeiss).

**Table 1:** Summary of immunostaining conditions.

Target	Antigen retrieval	Primary antibody	Secondary antibody
5-bromo-2-deoxyuridine (BrdU)	CB for 20 min at 105°C	Rat monoclonal IgG (ab6326; 1:400; Abcam, Cambridge, UK)	Biotinylated goat anti-rat IgG antibodies (1:400; Caltag, London, UK)
Single stranded DNA (ssDNA)	Not done	Rabbit polyclonal IgG (18731; 1:1000; IBL, Gunma, Japan)	Biotinylated goat anti-rabbit IgG antibodies (prediluted; SAB-PO kit, Nichirei, Tokyo, Japan)
E-cadherin	CB for 20 min at 105°C	Rabbit polyclonal IgG (sc-7870; 1:100; Santa Cruz Biotechnology, CA, USA)	Alexa Fluor 546 donkey anti-rabbit IgG antibodies (1:500; Life Technologies, Carlsbad, USA)
Foxl2	CB for 20 min at 105°C	Goat polyclonal IgG (ab5096; 1:500; Abcam)	Alexa Fluor 546 donkey anti-goat IgG antibodies (1:500; Life Technologies)
GATA4	CB for 20 min at 105°C	Goat polyclonal IgG (sc-1237; 1:100; Santa Cruz Biotechnology)	Alexa Fluor 546 donkey anti-goat IgG antibodies (1:500; Life Technologies)
Mast cell tryptase	CB for 20 min at 105°C	Mouse monoclonal IgG1 (ab2378; 1:400; Abcam)	Alexa Fluor 488 donkey anti-mouse IgG antibodies (1:500; Life Technologies)
c-kit	CB for 20 min at 90°C	Rabbit polyclonal IgG (sc-168; 1:500; Santa Cruz Biotechnology)	Alexa Fluor 488 donkey anti-rabbit IgG antibodies (1:500; Life Technologies)
DEAD (Asp-Glu-Ala-Asp) box polypeptide 4 (DDX4)	CB for 20 min at 90°C	Mouse monoclonal IgG (ab27591; 1:400; Abcam)	Alexa Fluor 546 donkey anti-mouse IgG antibodies (1:500; Life Technologies)

CB: 10 mM citrate buffer (pH 6.0)

### Transmission electron microscopy

The MRL/MpJ mouse ovaries at P0 and P7 were immediately fixed with 3% glutaraldehyde in 0.1 M cacodylate buffer (pH 7.3) at 4°C for 4 h. The ovaries were then fixed with 1% osmium tetroxide in 0.1 M cacodylate buffer at room temperature for 2 h, dehydrated with graded alcohol, and embedded in Quetol 812 (Nissin EM, Tokyo, Japan). Ultrathin sections were then stained with uranyl acetate and lead citrate, and were observed using a transmission electron microscope.

### Real-time RT-PCR

Total RNA from the ovaries and skin of MRL/MpJ mice was purified using TRIzol reagent (Life Technologies, Carlsbad, USA) and treated with DNase (Nippon Gene, Tokyo, Japan). Complementary DNA was synthesized from the RNA using ReverTra Ace (Toyobo, Osaka, Japan) and random primers (Promega, Madison, USA). Quantitative real-time PCR (qPCR) analysis was performed using gene-specific primers [24], Brilliant III Ultra Fast SYBR Green QPCR Master Mix (Agilent, Santa Clara, USA), and a real-time thermal cycler (MX 3000P; Agilent).

### Statistical analysis

The results were expressed as mean ± SEM values and were analyzed using nonparametric methods. The Mann-Whitney *U* test was used to compare between 2 groups. The Kruskal-Wallis test was used to compare among 3 or more groups, and multiple comparisons were performed using Scheffé's method.

## Results

### Spatiotemporal localization of MC in the female reproductive organs of neonatal MRL/MpJ mice

**Fig 1:**
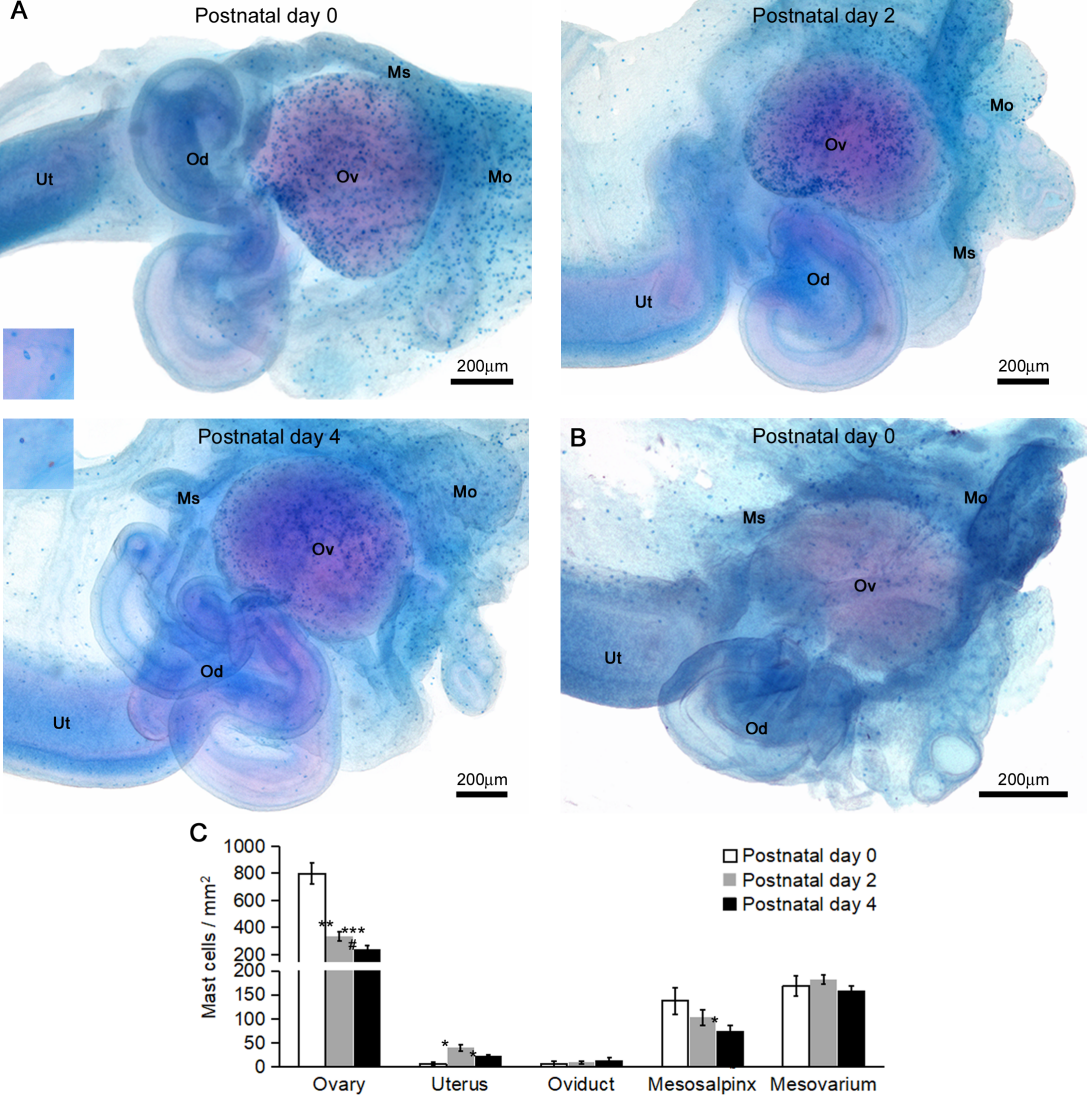
Mast cell distribution in the female reproductive organs of neonatal mice. (A and B) Whole-mount AB/SO staining of the female reproductive organs in perinatal MRL/MpJ mice (A) and C57BL/6N mice (B). Insets represent higher magnification showing mast cells. Mast cells are recognized as blue to purple dots. Me: mesosalpinx, Mo: Mesovarium, Od: oviduct, Ov: ovary, Ut: uterus. In all panels, left side represents the caudal region. (C) Mast cell densities in the female reproductive organs of neonatal MRL/MpJ mice. The mast cell densities were quantified by TB sections. Data represent mean ± SEM (n = 4–5 per group). * *P* <0.05, ** *P* <0.01, *** *P* <0.001 vs. postnatal day 0. # *P* <0.05 vs. postnatal day 2.

Firstly, we examined distribution changes of MCs in the female reproductive organs of MRL/MpJ mice (Fig 1A). To detect both of MMC and CTMC, AB/SO double staining was performed for whole-mount female reproductive organs because this staining visualizes both MMC- and CTMC-derived granules as blue and red, respectively [26]. At P0 to P4, the MCs distributed to the ovary as well as the mesosalpinx and mesovarium, but rarely to the oviduct or uterus. In C57BL/6N mice at P0, MCs localized to the mesosalpinx and mesovarium, but rarely to the ovaries (Fig 1B). Next, we quantified the MC densities in the female reproductive organs of neonatal MRL/MpJ mice using TB sections (Fig 1C). In the ovaries, abundant MCs were present at P0, and drastically declined to one fourth within 4 days after birth. Conversely, the MC densities were lower but stable at P0 to P4 in the uterus, oviduct, mesosalpinx, and mesovarium compared with the ovaries.

**Fig 2:**
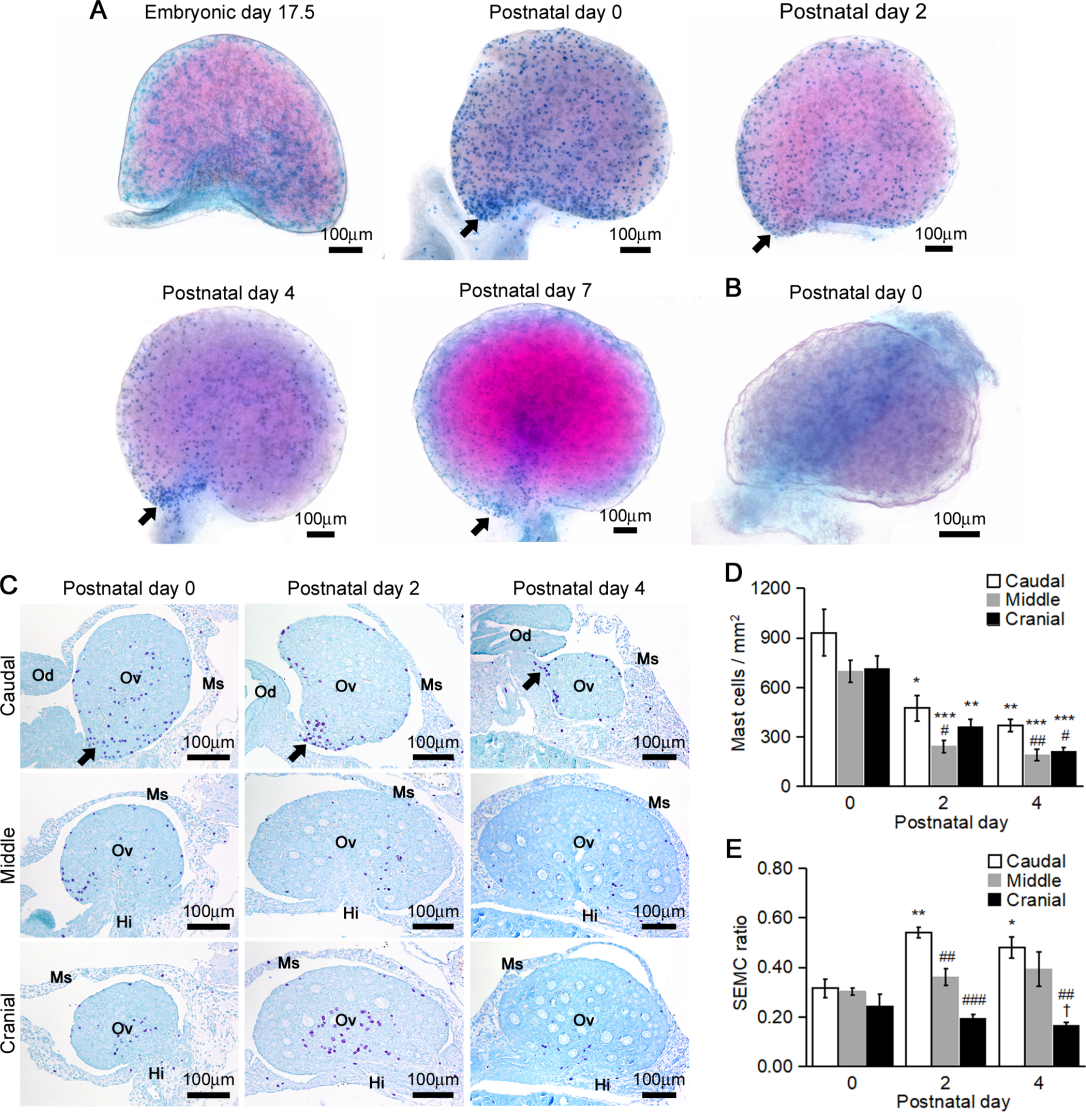
Mast cell distribution in the neonatal mice ovaries. (A and B) Whole-mount AB/SO staining of the ovaries in perinatal MRL/MpJ mice (A) and C57BL/6N mice (B). Mast cells are recognized as blue to purple dots. In all panels, left side and bottom side represents the caudal region and medial region, respectively. Arrows indicate the caudomedial region of the ovary where mast cells are densely concentrated. (C) Toluidine blue sections of the neonatal MRL/MpJ mice ovaries. Hi: hilum of the ovary, Me: mesosalpinx, Mo: Mesovarium, Od: oviduct, Ov: ovary. In all panels, bottom side represents the medial region. Arrows indicate the caudomedial region of the ovary where mast cells are densely concentrated. (D) Number of mast cells per total ovarian area. (E) Number of mast cells facing ovarian surface epithelium to that of total ovarian area (SEMC ratio). The mast cell densities were quantified by TB specimens Data represent mean ± SEM (n = 4–5 per group). * *P* <0.05, ** *P* <0.01, *** *P* <0.001 vs. postnatal day 0 among same region. # *P* <0.05, ## *P* <0.01, ### *P* <0.001 vs. caudal region among same age. † *P*<0.05 vs. middle region among same age.

Next, we examined the distribution changes of MCs in perinatal MRL/MpJ mouse ovaries by whole-mount AB/SO staining (Fig 2A). The number of ovarian MCs increased at P0 compared with E17.5, and decreased from P2 onward. Interestingly, although the MCs diffusely distributed in the ovaries at E17.5, they tended to change their localization to the caudomedial region of the ovary around the oviduct from P0 to P4 (Figs 1A and 2A). In C57BL/6N mice at P0, few ovarian MCs were observed in comparison with MRL/MpJ mice (Fig 2B). To further examine the distribution changes of the MCs to the caudomedial region of the ovary in MRL/MpJ mice, we quantified the MC densities and SEMC ratio in the caudal, middle, and cranial regions of the ovaries at P0 to P4 by using TB sections (Fig 2C–2E). The MCs majorly distributed to the caudal region of the ovaries compared with the middle and cranial regions throughout the experimental periods (Fig 2C). Although the ovarian MC density sharply declined with age in each region, it was higher in the caudal region than in middle and cranial regions at P2 and P4 (Fig 2D). The SEMC ratio in the caudal region increased at P2 and P4 compared with that at P0 (Fig 2E). Although the SEMC ratio did not differ among the ovarian area at P0, that in the caudal region was higher compared with the other regions at P2 and P4 (Fig 2E).

### Phenotypes of the ovarian MCs in MRL/MpJ mice

**Fig 3:**
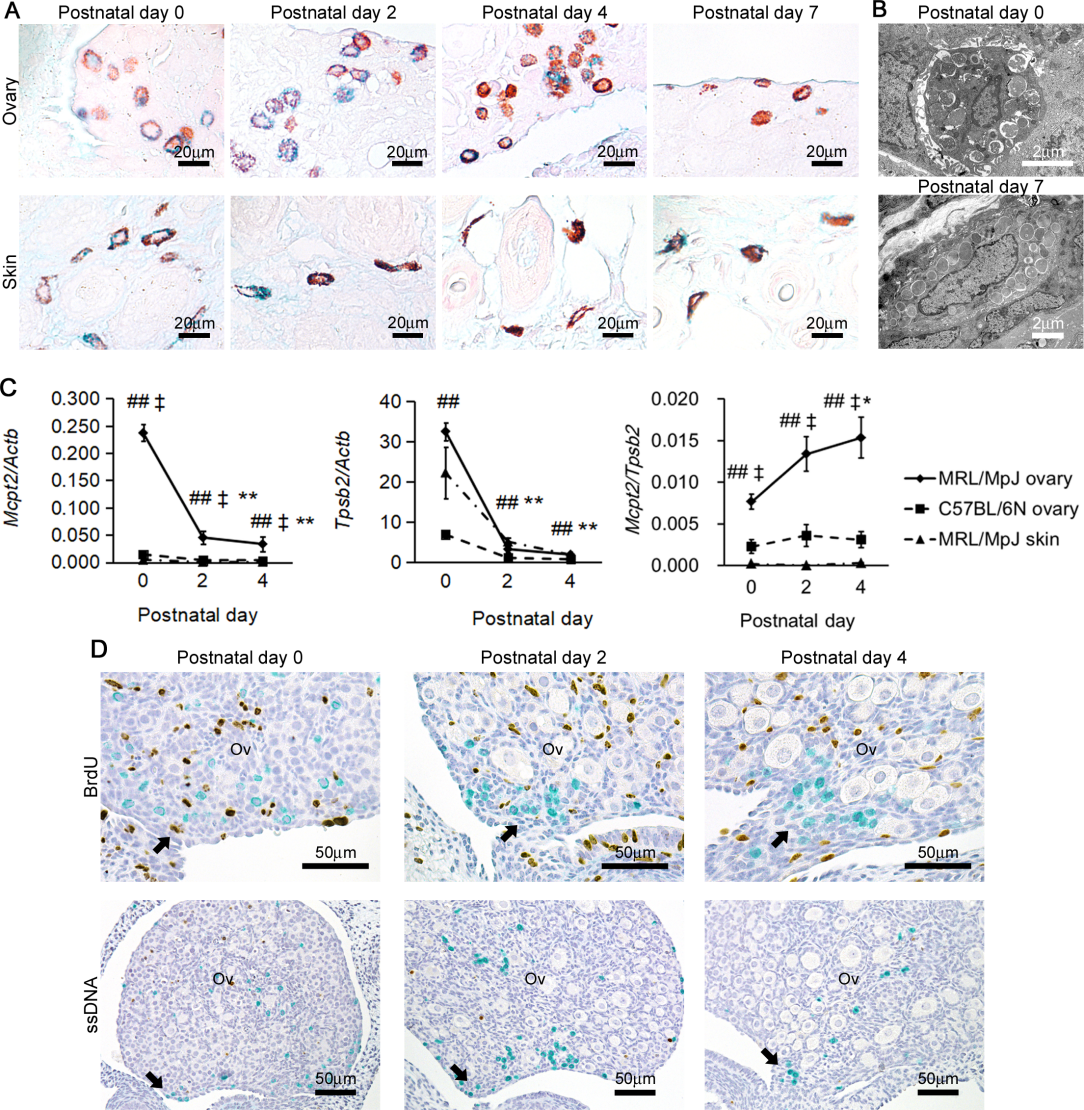
Phenotype of ovarian mast cells in neonatal MRL/MpJ mice. (A) AB/SO sections of neonatal MRL/MpJ mice. (B) TEM examination of neonatal ovarian MCs in MRL/MpJ mice. (C) Expression levels of MMC marker *Mcpt2*, CTMC marker *Tpsb2*, and *Mcpt2* normalized by *Tpsb2*. Data represent mean ± SEM (n = 4–5 per group).* *P* <0.05 vs. postnatal day 0 of the same organ. * *P*<0.05 vs. C57BL/6N ovary of the same age. ## *P*<0.01 vs. MRL/MpJ skin of the same age. (D) Immunohistochemistry for BrdU and ssDNA of MRL/MpJ mice ovaries. Ov: ovary. Arrows indicate the caudomedial region of the ovary where mast cells are densely concentrated. In all panels, bottom side represents the medial region.

In MRL/MpJ mice at P0, we have already reported that ovarian MCs possess CTMC phenotypes, however beneath SE they also express MMC marker [24]. Considering the morphological analysis as well, we predicted that MMC phenotypes were associated with their distribution changes. AB/SO staining of the neonatal MRL/MpJ mouse ovaries revealed that the ovarian MCs contained blue as well as red granules at P0 to P4, however the red granules tended to increase at P7 (Fig 3A). Although the skin MCs, typical CTMCs [3], had similar staining patterns as ovarian MCs, the former showed more elongated shapes than the latter. These results indicated that neonatal ovarian MCs showed intermediate characteristics of MMC and CTMC at P0 to P4, and the latter characteristics progressed at P7 (Fig 3A). TEM observation showed that more interstitial spaces were present around the ovarian MCs at P0 than those at P7 in MRL/MpJ mice (Fig 3B).

To examine the MMC phenotypes of ovarian MCs in more detail, we compared the expression levels of MMC marker *Mcpt2* and CTMC marker *Tpsb2* among the ovaries of MRL/MpJ and C57BL/6N, and MRL/MpJ mice skin where the MCs are classified as typical CTMCs [3] (Fig 3C). In consistent with the ovarian MC densities, the expression levels of *Mcpt2* and *Tpsb2* in MRL/MpJ mice ovary sharply decreased with time, but were higher than those in C57BL/6N mouse ovary and in MRL/MpJ mouse skin throughout the experimental periods. On the other hand, expression levels of *Mcpt2* normalized by *Tpsb2* tended to increase at P2 and P4 compared with P0 in the MRL/MpJ mouse ovaries, while they did not change with aging in the C57BL/6N mouse ovary and the MRL/MpJ mouse skin. Thus, these results indicated the relative increase of MMC phenotypes in MRL/MpJ mouse ovary at P0 to P4, although the number of ovarian MCs decreased.

To assess the effect of *in situ* cell proliferation or apoptosis in the distribution of ovarian MCs, the immunohistochemical localization of BrdU- or ssDNA-positive cells was examined, respectively. As a result, neither BrdU-incorporating nor ssDNA-positive MCs were found in the ovary at P0 to P4 (Fig 3D).

### Morphological characteristics of the MC-rich region of the MRL/MpJ mouse ovaries

**Fig 4:**
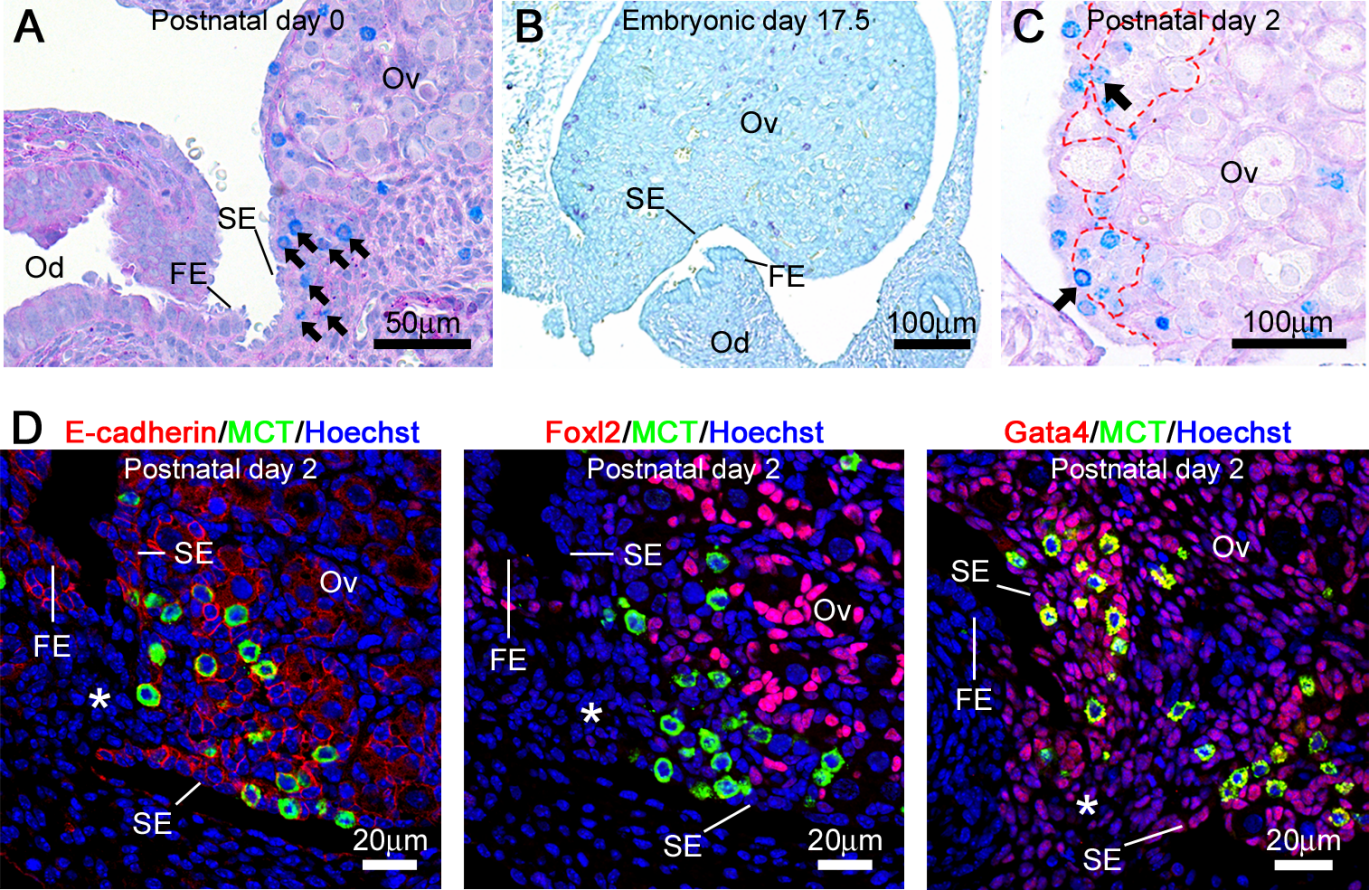
Characteristics of the MC-rich region of the MRL/MpJ mice ovaries. (A and B) Histology of ovary-fimbria connection in MRL/MpJ mice at postnatal day 0 by AB (pH 1.0)/PAS/hematoxylin staining (A), and embryonic day 17.5 by TB staining (B). (C) Ovarian section of MRL/MpJ mice at postnatal day 2. AB (pH 1.0)/PAS/hematoxylin staining. Dashed line represents the basement membrane. Arrows indicate mast cells present in the gap of basement membrane. (D) Expression patterns of E-cadherin, Foxl2, and Gata4 in the ovary-fimbria connection of MRL/MpJ mice ovaries at postnatal day 2. FE: fimbrial epithelium, MCT: mast cell tryptase, Od: oviduct, Ov: ovary, SE: surface epithelium. Asterisks indicate the ovary-fimbria connection. In all panels, bottom side represents the medial region.

We examined the histological characteristics of the caudomedial region of the ovaries shown in Fig 2. In this region, the columnar epithelium of the fimbria extended along the free border of the mesosalpinx, and then connected to the ovarian SE, which was called as the ovary-fimbria connection [27] (Fig 4A). While at E17.5, the oviduct did not connect to the ovaries, and the ovarian MCs had not accumulated around this region yet (Fig 4B). These results suggested that the ovarian MCs are densely localized after formation of the ovary-fimbria connection in MRL/MpJ mice. Interestingly, some MCs are localized at the gap of the basement membrane separating SE and oocyte nest from ovarian cortex at P2 (Fig 4C). Further, we examined the characteristics of the parenchymal cells surrounding MCs in the ovary-fimbria connection (Fig 4D, Table 2). E-cadherin was positive in the cytoplasm of oocytes, fimbrial epithelial cells, and surface epithelial cells. The expression of Foxl2 was restricted to the nuclei of follicular epithelial cells. Gata4 was positive in the nuclei of surface epithelial cells, follicular epithelial cells, and the ovarian parenchymal cells. The parenchymal cells around the ovary-fimbria connection were positive for E-cadherin and Gata4, but negative for Foxl2, which was consistent with the expression pattern of the surface epithelial cells (Table 2).

**Table 2:** Characteristics of the parenchymal cells in the ovary-fimbria connection.

Cell types	E-cadherin	Foxl2	Gata4
Oocytes	+	−	−
Surface epithelial cells	+	−	+
Follicular epithelial cells	−	+	+
Ovarian parenchymal cells	−	−	+
Fimbrial epithelial cells	+	−	−
Parenchymal cells in ovary-fimbria connection	+	−	+

+: positive, -: negative.

### Relations between oocytes and MCs around the ovary-fimbria connection in MRL/MpJ mice

**Fig 5:**
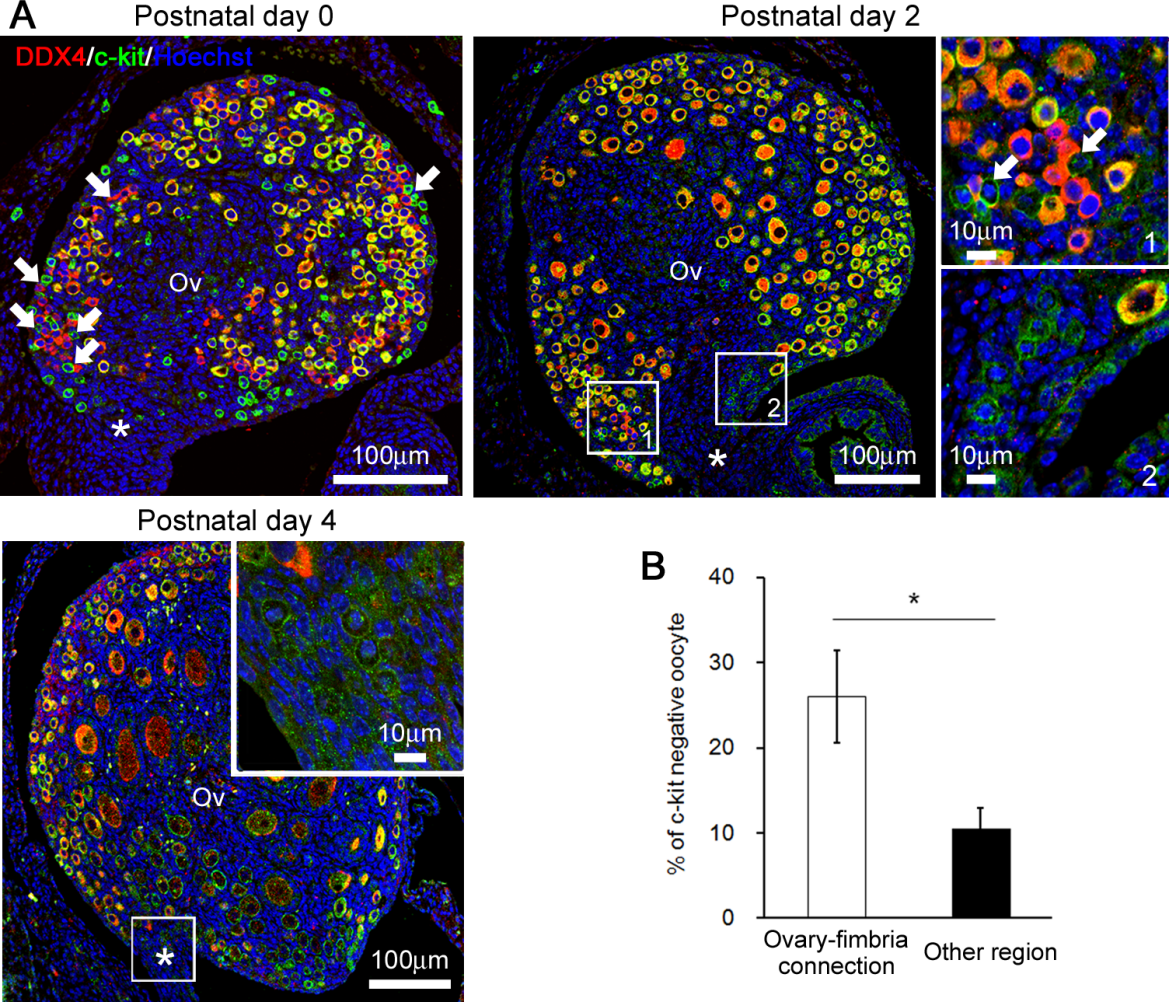
Oocytes around the ovary-fimbria connection in MRL/MpJ mice. (A) Immunofluorescence for DDX4 and c-kit in MRL/MpJ mice ovaries. Insets represent higher magnifications of boxed area. Arrows indicate direct contact between c-kit^+^ mast cells and DDX4^+^c-kit^-^ oocytes. Asterisks indicate the ovary-fimbria connection. (B) Ratio of DDX4^+^c-kit^-^ oocytes per DDX4^+^ oocytes in MRL/MpJ mice ovaries at postnatal day 0. Data represent mean ± SEM (n = 4).* *P* <0.05.

In MRL/MpJ mice at P0, the oocytes formed clusters called nests beneath the SE (Fig 5A). The majority of the DDX4^+^ oocytes were positive for c-kit, but some oocytes beneath the SE were negative (Fig 5A), indicating that the latter showed characteristics of immaturity [28]. Interestingly, more DDX4^+^/c-kit^-^ oocytes localized around the ovary-fimbria connection than in the other region of the ovary at P0 (Fig 5A and 5B). At P2, although almost all oocytes became primordial follicle beneath the SE, relatively small oocytes still formed nests around the ovary-fimbria connection, and some of them were still c-kit-negative (Fig 5A). The MCs, showing DDX4^-^/c-kit^+^, tended to accumulate adjacent to c-kit^-^ oocytes at P0 as previously reported [24], and they still contacted with the c-kit^-^ oocytes around the ovary-fimbria connection at P2 (Fig 5A). At P4, the oocytes developed to primordial and primary follicles, and the MCs rarely contacted with oocytes (Fig 5A).

## Discussion

The present study emphasized the importance of the ovary-fimbria connection in the distribution of the perinatal ovarian MCs. The number of the MCs is controlled by proliferation, survival, cell death, and migration [1]. In the neonatal MRL/MpJ mice ovary, the ovarian MCs showed neither proliferating nor apoptotic features, suggesting that the cellular migration majorly contributed to the localization changes of the postnatal ovarian MCs. In the adult uterus and ovary of mice and rats where the number of MCs vary over the estrous cycles, the MCs are morphologically heterogeneous by AB/SO staining, and the majority of AB-positive MCs depends on the estrus cycles [4, 5]. In rats treated with partial hepatectomy, the MMC marker (RMCP-2) expressing MCs increased in the regenerating liver [6]. These results suggest that the MMC phenotypes relate to the CTMC migration. We previously reported that the ovarian MCs possess CTMC phenotypes, however, the ones which localize beneath the SE express MMC marker as well in MRL/MpJ mice at P0 [24]. In the present study, the ratio of *Mcpt2* to *Tpsb2* increased with age and was higher than in the skin, and the ovarian MCs were intermediate type, between MMC and CTMC, as observed by AB/SO staining. Although immature CTMCs show MMC as well as CTMC phenotypes [6], the ovarian MCs differed in shape from the typical CTMC, the skin MCs in neonatal MRL/MpJ mice. These results further indicated that ovarian MCs possess MMC phenotypes in neonatal MRL/MpJ mice. Additionally, more interstitial spaces were present around ovarian MCs at P0, suggesting the absence of adhesive interactions with surrounding tissues which is generally required for leukocyte motility [29]. Some MCs were present at the gap of the basement membrane. Taken together, these results support the migration of the ovarian MCs along the SE and its association with MMC phenotypes.

In perinatal MRL/MpJ mice, the ovaries were the only female reproductive organ where the MCs densely localized. In the ovary, the MCs concentrated around the ovary-fimbria connection after birth. The parenchymal cells around ovary-fimbria connection showed the similar characteristics as the ovarian SE cells by the molecular expression patterns (E-cadherin^+^/Foxl2^-^/Gata4^+^). These results indicated that the neonatal ovaries, specifically, the ovary-fimbria connections and the SE played important roles in the MC migration. The ovary-fimbria connection is mixed with different microenvironments consisting of ovarian SE, oviductal epithelial cells, ovarian parenchymal cells, and oviductal parenchymal cells, thus functions as an ovarian cancer stem cell niche [27]. Maintenance of the stem cell niche and mast cell chemoattractant is carried out by several common molecules, such as stem cell factor (SCF) and C-X-C motif ligand 12 (CXCL12) [30–32]. These two cytokines are expressed in normal ovarian SE in human [33, 34]. In neonatal mice, although the expression of CXCL12 in the ovarian SE has been not reported, the ovarian epithelium as well as oocytes seems to express SCF [35]. Although we have demonstrated that the distribution of the ovarian MCs was controlled by chromosome 8, whose loci were designated as *mast cell in the ovary of MRL/MpJ 2*, indeed none of the MC chemoattractants were coded on the region [25]. In future, further studies are needed to elucidate the molecular mechanisms involved in the ovarian MC migration by using perinatal MRL/MpJ mice.

In the ovary of neonatal MRL/MpJ mice, MCs directly contact with degenerating oocytes in nests, thus might contribute to early follicular development [24]. In mice oocytes, the expression of c-kit decreases during fetal period, however re-appear at E18.5 during nest breakdown or later, suggesting that c-kit was a useful marker for oocyte maturation during perinatal period [28]. In neonatal MRL/MpJ mice ovary, the number of c-kit^-^ immature oocytes was higher around the ovarian fimbria than other region, suggesting that follicular development delayed around the ovary-fimbria connection. Importantly, the region was consistent with the MC-rich region, and some MCs contacted the c-kit^-^ oocytes in the nest at P2. These results might provide further evidence that the MCs played important roles on the early follicular development.

In conclusion, our results indicated that in postnatal MRL/MpJ mice, ovarian MCs migrated toward the ovary-fimbria connection which possessed similar characters to the ovarian SE. However, further studies are needed concerning the molecular mechanism for recruiting MCs by the ovary-fimbria connection. MRL/MpJ mice would be useful to elucidate the relations between neonatal immunity and reproductive systems.
